# The New Kid on the Block: HLA-C, a Key Regulator of Natural Killer Cells in Viral Immunity

**DOI:** 10.3390/cells10113108

**Published:** 2021-11-10

**Authors:** Sarah Vollmers, Annabelle Lobermeyer, Christian Körner

**Affiliations:** Leibniz Institute for Experimental Virology (HPI), Martinistraße 52, 20251 Hamburg, Germany; sarah.vollmers@leibniz-hpi.de (S.V.); annabelle.lobermeyer@leibniz-hpi.de (A.L.)

**Keywords:** HLA-C, NK cells, killercell immunoglobulin-like receptors, viral infection

## Abstract

The human leukocyte antigen system (HLA) is a cluster of highly polymorphic genes essential for the proper function of the immune system, and it has been associated with a wide range of diseases. HLA class I molecules present intracellular host- and pathogen-derived peptides to effector cells of the immune system, inducing immune tolerance in healthy conditions or triggering effective immune responses in pathological situations. HLA-C is the most recently evolved HLA class I molecule, only present in humans and great apes. Differentiating from its older siblings, HLA-A and HLA-B, HLA-C exhibits distinctive features in its expression and interaction partners. HLA-C serves as a natural ligand for multiple members of the killer-cell immunoglobulin-like receptor (KIR) family, which are predominately expressed by natural killer (NK) cells. NK cells are crucial for the early control of viral infections and accumulating evidence indicates that interactions between HLA-C and its respective KIR receptors determine the outcome and progression of viral infections. In this review, we focus on the unique role of HLA-C in regulating NK cell functions and its consequences in the setting of viral infections.

## 1. Introduction

The human leukocyte antigen (*HLA*) system represents a cluster of highly polymorphic genes that are associated with a large number of diseases. In its core function, the HLA system provides the underlying means for the immune system to distinguish between “self” and “non- or altered-self” [1]. Proteins encoded by *HLA* genes can be subdivided into two major groups, based on topography and function: HLA class I, comprising classical (HLA-A, -B and -C) and non-classical (HLA-E, -F and -G) molecules, and HLA class II. In particular, classical HLA class I molecules play a crucial role in inducing tolerance on the one hand and triggering innate and adaptive immune responses on the other. Being expressed by all nucleated cells, they represent the vehicles for the presentation of host-cell-, as well as pathogen-derived peptides which allow recognition by effector cells of the immune system [2]. The remarkable diversity of the *HLA* gene locus is the result of millions of years of evolution shaping the human species through natural selection. The tremendous impact of the *HLA* locus on the still-ongoing selection is reflected by the numerous disease associations between HLA and the outcomes of infections [3], autoimmunity [4], cancer [5], transplantation [6] and reproduction [7].

In the setting of viral infections, HLA class I molecules take a central position in a race between the effective elimination of virus-infected cells by the immune system and virus-mediated evasion from immune recognition. Cytotoxic T lymphocytes (CTLs) as well as natural killer (NK) cells are the major effector cells contributing to antiviral immunity, however utilizing opposing mechanisms for identifying virus-infected cells. CTLs recognize specific HLA:peptide complexes through their T cell receptor (TCR), whereas NK cells sense alterations of HLA class I surface expression and the presented peptides using germline-encoded receptors recognizing HLA class I. Most notably, CTLs require the presence of HLA class I for target cell recognition, whereas NK cells are triggered by the absence of HLA class I (“missing-self signal”) [8]. Viruses are challenged to counteract these opposing mode of actions and establishing a “sweet spot” in avoiding both CTL- as well as NK cell-mediated immune pressure. For this, viruses developed various evasion strategies that include modulation of HLA class I expression and antigen presentation.

In recent years, the classical HLA class I molecule, HLA-C, gained attention for its, previously neglected, role in viral immunity. In comparison to HLA-A and -B, HLA-C displays unique features. For one, HLA-C is the most recently evolved HLA class I molecule and only present in humans and great apes [9,10]. HLA-C is the only HLA class I molecule that is expressed on trophoblasts, which can be recognized by maternal decidual NK cells, and therefore represent a key molecule for the maternal-fetal immune tolerance in the establishment of pregnancy [11]. In contrast to its older siblings, HLA-C is expressed at a considerably lower level on the cell surface [12]. Hence, HLA-C was thought to play a minor role in the adaptive immune response of antigen-specific T cells [13]. For the functional maturation and induction of tolerance in NK cells; however, HLA-C is crucially involved by serving as the natural ligand for multiple members of the killer-cell immunoglobulin-like receptor (KIR) family [14]. Similar to HLA, the *KIR* gene locus is highly polymorphic and its gene products are predominantly expressed by NK cells [15,16,17]. KIRs interacting with HLA-C rapidly co-evolved with the first appearance of HLA-C and are able to recognize virtually all HLA-C allotypes, in contrast to the limited spectrum of HLA-A and -B allotypes recognized by human KIRs [18]. Finally, accumulating evidence showed that certain combinations of *HLA-C/KIR* alleles are associated with the clinical outcome of various types of diseases [19,20,21,22]. Given the impact of KIR/HLA-C interactions on the acquisition and progression of viral infections, this review sought to glean and discuss information on the unique role of HLA-C in regulating the immune responses of NK cells in the course of viral infections.

## 2. Structure, Expression, and Regulation of HLA-C

**Figure 1:**
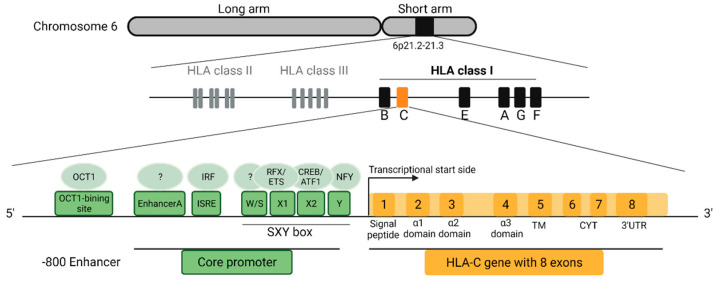
Location and structure of the *HLA-C* gene. HLA class I genes are located on the short arm of chromosome 6. The transcription of *HLA-C* is regulated by core promoter elements but also by distal regulators. The core promoter consists of the EnhancerA, ISRE and a SXY box. Compared to *HLA-A* and *-B*, the EnhancerA of *HLA-C* has no functional binding site for NFκB. ISRE activation is mediated through IFNγ stimulation which recruits the transcription factor IRF. The SXY box is composed of the W/S, X1, X2 and Y and is important for the binding of NLRC5 and formation of the enhanceosome. Transcription factors for W/S are still unknown, but X1 has binding sites for RFX and ETS, X2 has binding sites for CREB and ATF1 and Y for NFY. Moreover, the non-coding region of *HLA-C* contains an OCT1 binding site ~800 bp upstream of the core promoter region. *HLA-C* has 8 exons. Exon 1 encodes the signal peptide. Exon 2 and 3 encode the α1 and α2 domains which build the peptide binding grove. The α3 domain (exon 4) is connected to the transmembrane domain (TM) and cytoplasmic tail (CYT) (exon 5–7), anchoring the molecule to the cell membrane. Created with BioRender.com.

**Figure 2:**
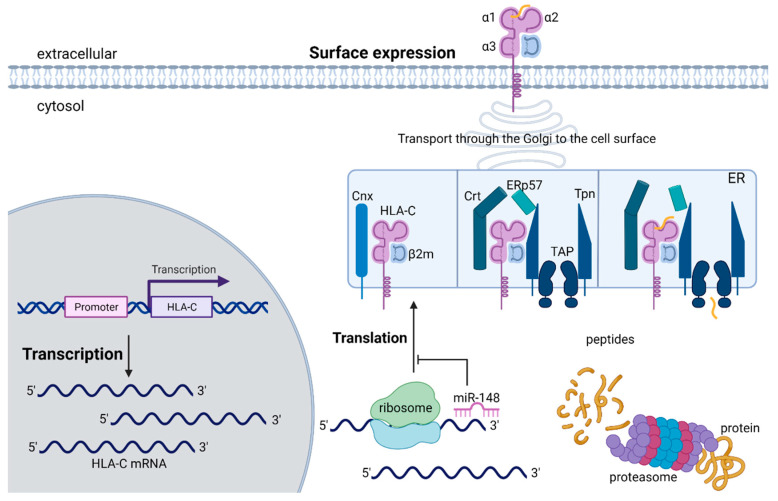
Protein synthesis pathway of HLA-C. Transcription of *HLA-C* is regulated through various transcription factors in the promoter region. Once the HLA-C mRNA is translated, the generated polypeptide undergoes proper folding, assembly and peptide loading. Translation of the HLA-C mRNA is regulated by micro-RNA-148 (miR148), which binds to the 3’ untranslated region. The assembly of the HLA-C heavy α-chain with β2m and a peptide is facilitated by a multi-subunit complex, composed of Cnx (calnexin), TAP (transporter associated with antigen processing), Tpn (type I transmembrane glycoprotein tapasin), the thiol oxidoreductase ERp57 and Crt (calreticulin). The HLA complex is loaded with high-affinity peptides that are generated by proteasome-mediated protein degradation in the cytosol. Peptides are transported into the ER and loaded onto the peptide binding grove. After peptide loading, the mature HLA-C:peptide complex dissociates from the multiprotein complex and is transported to the Golgi apparatus and then to the cell surface. Only HLA-C molecules with high-affinity peptides are transported to the cell surface to present the peptide to immune cells: Created with BioRender.com.

HLA class I molecules are heterodimers that consist of a glycosylated transmembrane heavy α-chain, encoded by chromosome 6 (6p21) and a light soluble non-covalently associated β2-microglobuline (β2m). Its encoding gene is located on chromosome 15 [23]. The promoter region of HLA class I molecules is highly conserved [24] and controls the transcription through regulatory elements that are located mainly in the proximal but also in the distal region upstream the transcriptional start side (Figure 1). The SXY box consists of W/S, X1, X2 and Y box motifs that are able to bind a range of transcription factors. The X1 box binds the RFX complex, which includes RFX5, RFXAP and RFXANK/B [25], whereas the X2 box interacts with CREB and ATF1 [26]. Moreover, the Y box is bound by NFY [27]. Factors for W/S binding are still unknown. The described complex is crucial for the recruitment of the transactivator NOD-like receptor caspase recruitment domain containing protein 5 (NLRC5) and the formation of an enhanceosome. NLRC5 is highly induced by IFNγ stimulation and also relevant for the expression of β2m [28,29]. In addition, the promoter region of HLA class I consists of an interferon stimulated response element (ISRE) and an EnhancerA, which are also important for cytokine-induced expression of HLA class I [30,31]. The production of IFNγ for example by activated lymphocytes mediates the expression and binding of interferon-response factors (IRFs) to ISRE via the JAK/STAT pathway. Compared to HLA-A with two functional NFκB binding sites and HLA-B with only one binding site [32], HLA-C exhibits none of these NFκB binding sites [30,33]. HLA-C encodes a polymorphic heavy chain, which can be subdivided into three domains: the two antigen-binding domains α1 and α2 and the α3 domain, which connects the molecule to the cell surface with a short cytoplasmic tail and interacts with the CD8 co-receptor of cytotoxic T cells [34,35]. The assembly of the heavy α-chain and β2m that occurs in the endoplasmic reticulum (ER) is tightly controlled and involves a number of co-factors (Figure 2). At least four of these accessory proteins thought to be involved in the assembly of the HLA class I/β2m heterodimers with peptides are the ABC transporter TAP (transporter associated with antigen processing), the type I transmembrane glycoprotein tapasin (tpn), ERp57 and calreticulin (Crt) [36,37]. This multi-subunit complex is referred to as peptide-loading complex (PLC). Prior to the incorporation into the PLC, the HLA class I heavy chain is associated with another ER resident co-factor, the lectin-chaperon calnexin (Cnx) [38,39]. ERp57, a thiol oxidoreductase together with Cnx plays an essential role in protein folding by promoting the formation of disulfide bounds [40]. Most newly formed heterodimers are unstable and need to enter the PLC in which Cnx is replaced with its orthologue Crt. The dissociation of HLA class I molecules from Cnx marks the end of the early assembly process. In the cytosol, proteins are degraded in the proteasome [41], transported into the ER lumen by TAP and loaded onto the peptide-binding grove of the HLA class I molecule. Only HLA class I molecules with high-affinity peptides are released from the PLC and enter the Golgi apparatus for subsequent glycosylation. Following this, the HLA:peptide complex is transported to the cell membrane where they present the endogenously-generated peptides to CD8^+^ T cells and NK cells [42].

Besides peptide specificity and binding affinity, the expression level on the cell surface is an important factor for an effective immune response. Compared to HLA-A and -B, HLA-C has a lower expression at the cell surface [23,43,44]. Several underlying mechanisms that are involved in HLA-C surface expression have been postulated and further investigated and include regulation at transcriptional, translational and post-translational levels. HLA-C mRNA has an increased turnover rate, which correlates with a low HLA-C cell surface expression [44]. HLA class I gene expression is cell type-dependent and can be induced by inflammatory cytokines [45,46]. The absence of NFκB binding sites consequently results in a weaker induction of HLA-C transcription by the inflammatory cytokines IFNγ and TNFα, which is associated with lower levels of HLA-C transcription compared to HLA-A and -B [26]. In addition, an SNP (rs2395471) in the OCT1 transcription factor binding site, located ∼800 bp upstream the HLA-C transcription start side, is significantly associated with HLA-C expression levels. Individuals with the rs2395471_A allele have higher HLA-C surface levels compared to individuals with the rs2395471_G. The higher binding affinity to the A allele results in a better promoter activation and higher expression level [47]. Another polymorphism (rs9264942) in the 5’ region of *HLA-C*, 35 kb away from transcription initiation is also associated with differences in HLA-C expression levels. A further recgulatory mechanism is the binding of microRNAs (miRNAs) to specific sites in the 3’ untranslated region (UTR). A variation (rs67384697) in the 3’ UTR of HLA-C affects the binding of miR-148. Alleles with an intact miR-148 binding site have a lower surface expression of HLA-C due to the binding of the miRNA, which results in an inhibition of protein expression. Alleles with a deletion at position 263 downstream of the HLA-C stop codon are able to escape the post-transcriptional regulation because of the loss of the miR-148 binding site [48]. HLA-C heavy chains and β2m assembly is less effective and slower, which leads to an accumulation of β2m-free heavy chains [43]. HLA-C is more selective in its presentation of antigens because of its restricted repertoire of peptides, characterized by a lower affinity [49]. This leads to an accumulation of HLA-C molecules, which are rapidly cleared out of the ER. In line with that, variations in exon 2 and 3, encoding the peptide-binding site domains, contribute to differential cell surface expression. Comparison of two *HLA-C* alleles that have high (HLA-C*05) and low (HLA-C*07) expression levels demonstrated that the peptide-binding groove of HLA-C*05 is more permissive and filled with large aromatic residues, which allows the binding of a large range of distinct peptides [50]. The regulation of HLA-C expression on NK cells itself is an important factor for NK cell function and differentiation, supporting the evolutionary development of HLA-C primarily for controlling NK cell function. An SNP in the ETS-binding site of an NK cell-specific promoter element of *HLA-C* results in different HLA-C expression, influencing NK cell activity. The disruption of the ETS site results in reduced transcript levels and lower HLA-C expression, which increases NK cell activity [51]. Taken together, the expression of HLA-C is controlled by distinct mechanisms affecting transcription, translation and post-translation. HLA class I molecules share the same core promoter elements, but, compared to HLA-A and -B, HLA-C exhibits regulatory elements that lead to a lower cell surface expression.

## 3. Regulation of NK Cells by HLA-C

Like other classical HLA class I molecules, HLA-C is capable of regulating CTL activity through interactions between the TCR and HLA:peptide complexes. Given the low surface density of HLA-C, in contrast to HLA-A and HLA-B, HLA-C is considered to play a minor role in triggering the adaptive immune system [23,52]. However, for several infectious diseases, HLA-C restricted CTLs have been described [53,54]. Therefore, the overall impact on disease control is still debated.

### 3.1. Killer-Cell Immunoglobulin-Like Receptors (KIRs) Recognize HLA-C

The other major group of interaction partners of HLA-C are members of the KIR family. KIRs are predominantly expressed on mature NK cells, acting as key regulators of development, tolerance and activation, but are also expressed on a subset of T cells [55,56]. Like HLA class I molecules, the *KIR* gene family is characterized by an extraordinary high degree of genetic and functional diversity, resulting in varying susceptibilities to pathogens and diseases. The diversity arises from variability in *KIR* gene content, *KIR* gene copy numbers and from allelic polymorphism [17,57]. The *KIR* gene family is located on chromosome 19q13.4 and consists of up to 15 genes. They are similar in structure but show varying features in terms of expression, signaling pathways and ligand specificity [58]. The KIR nomenclature is based on the number of extracellular domains (2D or 3D) and on the length of the cytoplasmic tail [S (short) or L (length)], reflecting the function of the encoded protein (activating or inhibitory) [59]. While the inhibitory forms function via immunoreceptor tyrosine-based inhibitory motifs (ITIMs), activating types possess truncated cytoplasmic domains lacking ITIMs [60]. These molecules associate with adapter molecules that contain immunoreceptor tyrosine-based activation motifs (ITAMs).

The *KIR* gene content can be separated into two haplotypes, A and B. Beside the frame work genes *KIR3DL3*, *KIR3DP1*, *KIR2DL4* and *KIR3DL2*, present in almost all individuals, A haplotypes have a fixed gene content, comprising the pseudogene *KIR2DP1*, and additionally encodes the inhibitory receptors KIR2DL1, KIR2DL3, KIR3DL1, KIR3DL2 and only one activating receptor, KIR2DS4. Haplotype B is enriched for activating KIRs (*KIR2DS1/2/3/5* and *KIR3DS1*) and the inhibitory receptors, *KIR2DL2* and *KIR2DL5* [61]. Individuals expressing haplotype A are thought to exhibit an improved response to pathogens, whereas B haplotypes correlate with improved reproductive fitness [62,63,64]. The current *KIR* genes and the resulting haplotypes display a snapshot of the rapid evolution of the *KIR* gene locus. The two haplotypes are thought to be maintained within the human population by balancing selection. However, the frequency of these haplotypes varies significantly between populations [65,66]. The close proximity of the *KIR* genes and their organization in the *KIR* locus probably facilitated gene expansion by duplication and recombination, and is reflected by the substantial linkage disequilibrium between KIRs [67].

**Figure 3:**
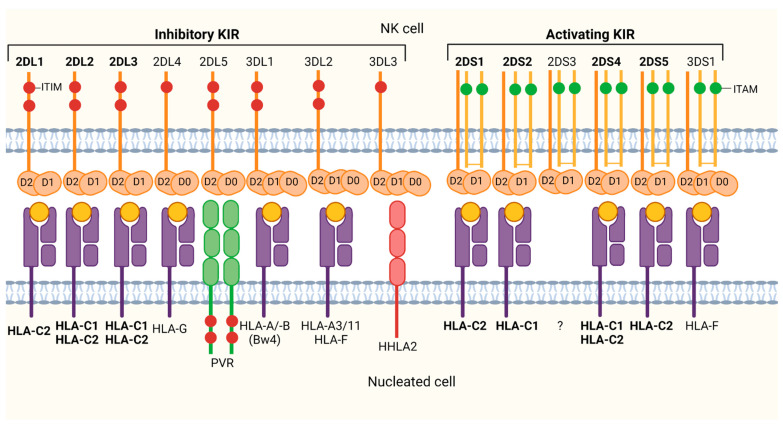
KIR family and respective HLA class I ligands. Illustration of the structure and distribution of inhibitory and activating KIRs and their respective ligands. Killer-cell immunoglobulin-like receptors (KIR) (orange), expressed predominantly on NK cells, interact mainly with HLA class I molecules (purple) and its presented peptides (yellow). Each KIR exhibits a specificity for only a selection of HLA class I molecules, including HLA-C allotypes (bold). For example, inhibitory KIR2DL1 and activating KIR2DS1 exclusively interact with HLA-C2, while the inhibitory receptors KIR2DL2/L3 are cross-reactive for the HLA-C1 and -C2 allotypes. Inhibitory KIRs (and the poliovirus receptor (PVR)) carry immunoreceptor tyrosine-based inhibitory motifs (ITIM, red circles), signaling NK cell inhibition upon receptor-ligand engagement, whereas activating KIRs associate with adapter molecules that contain immunoreceptor tyrosine-based activation motifs (ITAMs, green circles) conferring an activating signal. Created with BioRender.com.

With a few exceptions, HLA class I molecules represent the primary natural ligands of both activating and inhibitory KIRs (Figure 3). However, each receptor has a specific spectrum of HLA class I ligands. Notably, only a limited number of HLA-A and -B allotypes serve as KIR ligands, whereas virtually all HLA-C allotypes serve as interaction partners for one or more KIRs [18], consistent with HLA-C being evolved to be a superior and more specialized ligand for KIRs [68]. HLA-C allotypes are selectively recognized by seven different inhibitory and activating KIRs: KIR2DL1, KIR2DL2, KIR2DL3, KIR2DS1, KIR2DS2, KIR2DS4 and KIR2DS5. Their ligand specificity is determined by multiple factors comprising the extracellular domains of both, HLA-C and KIRs, as well as the presented peptide pool. KIR recognition of HLA-C is impacted by a dimorphism at position 80 of the α1 domain of HLA-C: The C1 epitope is defined by asparagine (N) and the C2 epitope is characterized by lysine (K) (Table 1) [69]. Inhibitory KIR2DL1 and activating KIR2DS1 [70,71], which carry a methionine at position 44 and some KIR2DS5 allotypes [72], exclusively recognize the HLA-C2 epitope. Inhibitory KIR2DL2, KIR2DL3 [71] and activating KIR2DS2 allotypes, which have a lysine at position 44, exhibit a selective affinity for HLA-C1 epitopes. Additionally, certain KIR2DL2 and KIR2DL3 allotypes are also cross-reactive with selected HLA-C2 allotypes [71,73]. Similarly, activating KIR2DS4 interacts with some HLA-C1 and -C2 allotypes [74]. Many activating receptors evolved from their inhibitory counterparts [75], thereby displaying a high degree of sequence homology in their extracellular Ig domains. This evolutionary relationship is also reflected by their similar binding specificities. However, inhibitory KIRs exert higher avidity for their respective HLA class I ligands than their activating counterparts [76]. While the discrimination of C1 and C2 is defined by the dimorphism at position 80 of HLA-C, [77], specificity and avidity of KIRs for HLA-C are strongly impacted by polymorphisms in key positions [78]. For example, KIR2DS1 and KIR2DL1 differ by only seven amino acids in their extracellular portion and, nonetheless, KIR2DS1 is known to bind about 50% less pronounced to HLA-C2 than its inhibitory counterpart KIR2DL1 [71,79]. KIR/HLA-C interactions, referring to specificity, affinity, as well as avidity is influenced by the specific KIR allotypes [80]. Binding affinities between different KIR and HLA-C allotypes show huge differences, which are important for the prediction of the NK cell response upon HLA class I ligand recognition by KIRs. Furthermore, binding of specific KIRs to their respective HLA ligands can be modulated by the presented peptide [81,82]. The loaded peptide is essential for correct folding, expression and function of HLA class I molecules. The HLA class I-presented peptide repertoire plays a significant role in KIR binding and NK cell function and, furthermore, influences the response of NK cells against certain viral infections [19,83]. KIRs are sensitive to changes in the peptide content presented by HLA class I. KIR2DL3^+^ NK cells for example, are suggested to be more sensitive to changes in the peptide content of the HLA class I binding groove than NK cells expressing other KIRs [84]. Several studies have shown that miRNAs regulate the expression of genes that are involved in the effector functions of NK cells [85]. The microRNA miRNA-146a-5p modulates the expression of KIR2DL1/L2 by interacting with the 3’UTR of the mRNA. Moreover, in silico functional characterization identified among others HLA-C as a putative target of miRNA-146a-5p [86]. Overall, HLA-C allotypes, being entirely recognized by KIRs, and the peptide presentation influencing KIR/HLA-C interaction leads to HLA-C having an outstanding role among the classic HLA class I molecules.

### 3.2. Appearance of HLA-C Triggered Rapid Co-Evolution of HLA-C Recognizing KIRs

The *KIR* gene cluster shows extensive genetic diversity, only exceeded by the *HLA class I* loci. The extreme variability of the *KIR* and *HLA* gene loci is thought to provide protection against a wide variety of pathogens, with different KIR/HLA combinations leading to protection against distinct diseases and to reproductive success. Low-resolution analysis showed that KIR and their HLA ligands have evolved in concert across populations worldwide [88]. The extensive diversity of the *HLA* and *KIR* gene loci and the central role of their interactions in modulating immune responses are presumed to favor the co-evolution of genotypic combinations of these two loci in order to maintain appropriate functional interaction. Furthermore, evidence of co-evolution has been suggested in disease studies [65,89,90], as well as in comparative genetic studies across primate species. Co-evolution was observed for example in Old World monkeys. Rhesus macaque comprise ligands for HLA-A and -B, but not for HLA-C [91]. An abundance of *HLA-A* and *-B* genes that encode the Bw4 epitope is accompanied by a corresponding expansion of the respective lineage II KIR [92,93,94]. In contrast, the organization of the orangutan and chimpanzee *KIR* loci is inverted. Corresponding with the emergence and fixation of HLA-C, the centromeric region of the KIR locus contains different combinations of nine lineage III KIR genes encoding receptors that recognize the C1 or C2 epitopes [68,95], while the telomeric region comprises only one lineage II KIR encoding a receptor for Bw4-like epitopes of HLA-A and HLA-B. Altogether, HLA-C developed under natural selection in the higher primates to be a more specialized ligand for KIRs than either HLA-A or HLA-B [68]. Being absent in Old World monkeys, extravillous trophoblasts of hominids express HLA-C but not HLA-A or HLA-B during pregnancy, correlating with the emergence of HLA-C in the orangutan [96]. A gender bias in terms of non-random associations between KIR core haplotypes and HLA class I has been found in the Japanese population [97]. Several associations between *KIR* and *HLA* genes were limited to females supporting the view that reproduction is a strong selective pressure acting on *KIR* genes [62,65,97,98].

Nevertheless, analysis of co-evolution remains complex: first, *KIR* and *HLA* genes are inherited on different chromosomes, second not all functional interactions have been defined and third known interactions are epistatic, meaning that the presence of genes or alleles encoding corresponding receptor–ligand pairs is necessary for functional activity, but the presence of one without the other has no influence on effector cell activity. Due to that, direct evidence from human population studies pinpointing receptor-ligand combinations that are major factors in their co-evolution is lacking. Observations of significant correlations between frequencies of specific *KIR* genes and *HLA* alleles encoding their corresponding ligands would support the idea of these unlinked loci co-evolving.

### 3.3. HLA-C Regulates NK Cell Activity through Inhibitory KIRs

The engagement of KIR receptors by HLA-C leads to intracellular signaling in NK cells. Activating KIRs contain a positively charged amino acid in the transmembrane domain, which allows the recruitment of the adapter molecule DAP12 that comprises an activating ITAM [99]. Inhibitory KIRs comprise ITIM in their cytoplasmic tail that transfer the signal to the cell, once tyrosines in the ITIMs become phosphorylated and associate with intracellular phosphatases, such as SH2-domain-containing protein tyrosine phosphatase 1 (SHP-1) [100]. This provides a strong inhibitory stimuli that is able to overwrite activating signaling in NK cells [101]. In addition, NK cells undergo a process of functional maturation that requires the interaction of inhibitory *KIR* with *HLA* class I alleles expressed by the host. This process, necessary for calibration of NK cell function, enabling “self” vs. “non- or altered-self” discrimination, is termed education. To date, there are three different models that describe how NK cell education is achieved: “Licensing/arming”, “disarming”, and “tuning” [102]. Studies in the field of immunometabolism suggest that NK cell metabolism might play a role in education as well as they revealed that cellular metabolism is able to shape immune cell effector functions [103,104]. Apart from the current advances, the molecular strategies of education are not yet fully understood, partially because the educational process of T cells proceeds differently. NK cell education is mediated through the engagement of inhibitory KIRs, consequently called self-inhibitory receptors with their cognate self-HLA class I molecules on healthy host cells-thus mediating self-tolerance and preventing NK cells from killing healthy cells [105]. Education leads to the maturation of a functionally competent NK cell repertoire that is adapted to the HLA class I molecule environment of the host [106]. Due to the stochastic expression of many inhibitory receptors, an individual NK cell expresses either none, one or various self-inhibitory receptors. Educated NK cells exhibit a higher sensitivity against HLA class I molecule-induced inhibition and are characterized by a low activation threshold with regard to target cells with modulated or lower HLA class I molecule expression like virus- infected cells [107]. Uneducated NK cells, on the other hand, are weakly or non-reactive to HLA class I molecule-negative target cells. The education of NK cells allows rapid recognition of changes in HLA class I expression and reaction by NK cells with increased sensitivity [108].

Collectively, HLA-C plays a central role in the education process to establish self-tolerant NK cells. From an evolutionary perspective, it is particular important as the most prominent ligand for KIRs.

## 4. HLA-C-Mediated Impact of Viral Immune Response

An increasing number of studies investigated the impact of KIR/HLA-C interactions on the outcome and progression of viral infections and further explored the underlying mechanisms. Genome-wide association studies (GWAS) provide an important first insight whether host genetics may impact the course of infection. Ex vivo and in vitro assessment of the NK cell repertoire, including NK cells expressing HLA-C recognizing KIR, and its antiviral activity allow an inference on the contribution of those subsets to viral control. In vitro infection models allow the identification of immune evasion strategies including ones that specifically demonstrate NK cell-mediated immune pressure and viral escape through the selection of peptidevariants. The main findings of these topics are summarized in Table 2 for HIV-1, HCV and CMV infections.

### 4.1. Human Immunodeficiency Virus (HIV)

Among HIV-1-infected individuals, a small group (0.15 to 2.5%) is able to intrinsically control viral load without antiretroviral therapy. These HIV controllers are defined by a durable low threshold viral loads, stable CD4^+^ T cell counts and lower risk of transmission to others [109]. Many of these individuals have protective *HLA* alleles and potent T cell responses, facilitating control of viral replication [110,111]. However, there are subgroups of HIV-1 controllers without protective *HLA* alleles or strong T cell response suggesting that additional host factors are relevant for HIV control [112]. In addition, case studies have shown that an effective NK cell response may contribute to early control of HIV-1 replication [113,114]. GWAS comparing HIV controllers and chronically infected individuals with advanced disease progression highlighted the importance of HLA-C in HIV-1 infection [115,116]. Two independent polymorphisms have been identified that are associated with HLA class I. One polymorphism (rs2395029) in the HLA complex P5 (HCP5), 100 kb centromeric from *HLA-B,* is associated with the HLA-B*57:01 allele. HLA-B*57:01 is known to have a protective impact on HIV-1 progression, linked to restriction of HIV-1 replication, non-progressive disease [117] and lower viral loads [118]. The second most significant polymorphism (rs9264942) is located 35 kb (-35C/T) away from the transcription start site of *HLA-C* and explains 6.5% of the total variation in HIV-1 set points. Individuals with the -35C allele have higher CD4^+^ T cell counts and HLA-C mRNA levels [119,120]. They also exhibit a higher HLA-C cell surface expression, progress more slowly to AIDS and control viremia significantly better than individuals carrying *HLA-C* alleles expressed at lower levels [115,121,122].

Characterization of cell surface expression levels of common HLA-C allotypes showed a significant association of HLA-C expression levels and HIV control. High HLA-C expression levels are associated with increased likelihood of HLA-C-restricted cytotoxic T cell response [123] and increased frequency of mutations in HLA-C-presented HIV-1 epitopes [124]. Another factor that is connected to HLA-C expression is an SNP in the binding site of the miR-148 in the 3’ untranslated region of HLA-C. The polymorphism at position 263 (263I/D) in this region leads to a different expression of various HLA-C allotypes. Binding of this miR-148 leads to an inhibition of the *HLA-C* allele and low cell surface expression. Individuals with at least one copy of a miR-148-inhibited allele showed significant effects of miR-148 expression levels in HIV-1 control [48]. HLA-C expression levels are also affected by miR-148 expression levels itself. An SNP (rs735316) downstream of the 3’end of the mature miR-148 sequence is associated with miR-148 expression level also affecting the HLA-C expression and level of HIV-1 control [125].

**Figure 4:**
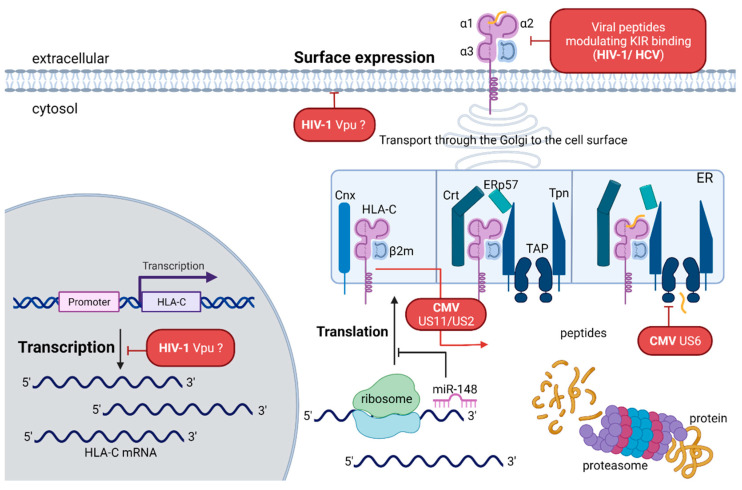
Modulation of HLA-C by viral proteins. Viruses utilize multiple mechanisms to evade immune recognition, including downmodulation of HLA class I molecules or selection of specific peptide variants. Viral escape mechanisms can influence HLA-C expression on the transcriptional, translational, post-translational and protein level. HIV-1 is able to decrease HLA-C surface expression by specific variants of the accessory protein Vpu. CMV encodes different US proteins that target HLA-C heavy chains for proteasomal degradation or block the transport of peptides into the ER. Moreover, HIV-1 and HCV are able to select for specific HLA-C-restricted peptides that modulate the activation of NK cells by altering KIR binding. Created with BioRender.com.

As NK cells directly interact with HLA class I molecules and are known for killing virus-infected cells quite efficiently, studies have shown that specific KIR/HLA haplotypes [126,127,128] and in detail specific combinations of HLA-C and its corresponding KIR receptors, have an impact on anti-HIV immunity and clinical outcome. KIR^+^ NK cells can exert immunological pressure on HIV-1. In turn, HIV-1 is able to evade this immune pressure by selecting for KIR2DL2-associated amino acid polymorphisms, which enhance the binding of the inhibitory KIR to HIV-1-infected cells and reduce the antiviral activity of these KIR^+^ NK cells [83]. NK cells in primary HIV-1 infections showed a higher frequency of KIR2DL1-3 in the presence of their cognate HLA-C ligand compared to healthy individuals. KIR2DL1-3^+^ NK cells were more polyfunctional in primary HIV-1 infection in individuals with their cognate HLA-C haplotypes; however, they were disproportionately subject to NK cell dysfunction in the transition to the chronic phase of infection [129]. Genotyping of *HLA* and *KIR* in chronically infected and antiretroviral-free HIV-1-infected individuals from Japan revealed a protective effect of KIR2DL2/HLA-C*12:02 and KIR2DL2/HLA-C*14:03 genotypes. Both combinations correlated with lower plasma viral load [130]. A study of a South African cohort of chronically HIV-1-infected ART-naïve adults reported a deleterious effect of the HLA-C*16:01/KIR2DL3^+^ pair in HIV-1 clinical outcome [131]. In addition, two other studies observed a deleterious effect of *KIR2DL3/HLA-C1* on HIV-1 outcome [132,133], but others reported a protective effect against mother-to-child transmission [134] and HIV-1 infection in exposed uninfected intravascular drug users [135]. HIV-1 and also other pathogens are able to decrease HLA class I expression to avoid the presentation of viral peptides and thus activating cytotoxic CD8^+^ T cells. The HIV-1 accessory protein Nef specifically downmodulates HLA-A and -B on infected CD4^+^ T cells, whereas HLA-C is not affected by Nef [136,137,138]. Based on these findings, established models proposed that HIV-1 does not regulate the HLA-C expression to protect the infected cell against the innate immune response of NK cells through the interaction of HLA-C with inhibitory KIR2DL receptors [139]. However, a 2016 study by Apps et al. demonstrated that many primary HIV-1 clones are able to downregulate HLA-C to a different extent, and that this is mediated by the HIV-1 accessory protein Vpu. The reduction of HLA-C from the cell surface impairs the ability of HLA-C-restricted cytotoxic T cells to suppress viral replication. The dynamic regulation of HLA-C by HIV-1 provides an opportunity to react to different immune pressures trigged by either dominant NK cell or CD8^+^ T cell responses. The characterization of primary HIV-1 viruses revealed an adaption of Vpu-mediated downmodulation of HLA-C to the host HLA genotype [140]. NK cells are able to sense changes in HLA-C expression by an increased antiviral activity when exposed to HIV-1-infected CD4^+^ T cells with different abilities to downmodulate HLA-C from the cell surface [141]. *HLA-C* alleles with high surface expression levels showed an association with strong viral downregulation of HLA-C [142]. In a recent publication, Hopfensperger et al. showed that HIV-2, which lacks *vpu*, is able to downmodulate HLA-C surface expression by the accessory protein Vif and that the decreased surface expression is associated with higher killing of infected cells by NK cells [143]. Vpu targets HLA-C at the protein level, independently of its ability to suppress NFκB-induced gene expression [142,143,144]. Due to a putative NFκB binding site upstream the HLA-C core promoter [47], it is also possible that Vpu inhibits HLA-C mRNA expression [143] (Figure 4).

Although multiple studies showed that HLA-C and inhibitory KIR interactions have an impact on disease progression and NK cells are primarily known to be triggered by the missing-self signal, there is increasing data that viral peptide-presentation of HLA-C can also modulate NK cell function by different mechanisms. Functional analysis of HLA-C*01:02-restricted HIV-1 p24 Gag epitopes showed that certain epitopes can modulate binding to KIR2DL2 and subsequently NK cell function [145]. Sequence polymorphisms in p24 Gag enabled improved binding of KIR2DL2/3 to HLA-C*03:04 expressing cells, resulting in inhibition of these NK cells [19,146]. Functional analysis of HIV-1-derived peptides and HLA-C*14:03^+^ and HLA-C*12:02^+^ cells showed a reduced expression of the HLA-C:peptide complex on the surface of HIV-1-infected cells, which consequently had an impact on NK cell recognition and activation without changing the binding affinity between the KIR receptor and the HLA:peptide complex [130]. A recent study from Ziegler et al. observedthat HIV-1 infection induced changes in HLA-C*03:04-presented peptides, which reduced the binding of KIR2DL3 receptors and led to an enhanced recognition of HIV-1-infected cells by NK cells [147]. Taken together, not only a potent T cell response and protective *HLA* alleles are involved in HIV-1 control, but also the sensitive network of HLA-C surface expression, KIR binding and NK cell activation contribute to an effective viral immune response and underscore the sensitive balance between innate and adaptive immune response upon HIV-1 infection.

### 4.2. Hepatitis C Virus (HCV)

NK cells contribute to the immune response in HCV infection [148]. Several studies showed that KIR/HLA-C interactions impact the outcome of HCV infection and are linked to spontaneous resolution of HCV infection. Khakoo et al. observed that the combined presence of *KIR2DL3* and *HLA-C* alleles encoding for its ligand HLA-C1 directly affected the resolution of HCV infection in Caucasians and African Americans. Individuals homozygous for *HLA-C1* alleles were enriched in the group with resolved infections compared to the group of individuals with persistent infection. The protective association of *HLA-C1/C1* was only significant in individuals homozygous for *KIR2DL3* but not for individuals homozygous for *KIR2DL2* or heterozygous for *KIR2DL2/L3* [20]. Moreover, the frequency of *KIR2DL3* homozygosity in combination with *HLA-C1* was shown to be higher in seronegative aviremic individuals as compared to individuals with chronic HCV, indicating a benefit of this specific *KIR2DL3-HLA-C1* combination for the outcome of HCV infection [149]. Males, but not females, carrying *KIR2DL2* and *KIR2DS2* genes had a 1.7 higher probability to become chronically infected with HCV than males lacking these genes [150]. Therapies for HCV, including pegylated interferon (PegIFN) alpha plus ribavirin (RBV), can achieve a sustained virologic response (SVR) of 40–50% in patients infected with the most common viral genotype 1 (GT1) [151]. The success of the treatment also relies on host and viral factors. Genotyping of *HLA-C* and *KIR* in patients with chronic HCV GT1 infection with PegIFN/RBV treatment-induced clearance and treatment failure showed that the *HLA-C2* homozygous genotype was more frequent in patients that do not respond to the treatment (NSVR) [152]. Analysis of *KIR2DL2/L3* alleles in chronic HCV-infected patients revealed that the homozygous *KIR2DL3-HLA-C1* genotype was more frequent in patients with SVR than in NSVR. In contrast, *KIR2DL2/L2-HLA-C1/C2* was more common in NSVR patients [153].

A study of 125 individuals with chronic HCV in Brazil showed a higher frequency of *KIR2DL2* and *KIR2DL2/HLA-C1* genes in these individuals and an association of *KIR2DS2*, *KIR2DS2-HLA-C1* and *KIR2DS3*, independent from *KIR2DL2*, but they did not observe any correlation with therapy response [154]. Besides reports of the beneficial *KIR2DL3/HLA-C1/C1* genetic association with HCV outcome, there are studies that did not confirm this association or described other *KIR/HLA* combinations that may influence the outcome of HCV infection. Patients with persistent infection had a higher frequency of *KIR2DL3* and a lower frequency for *KIR2DL2* compared to individuals who cleared the infection [155]. A recent study of HIV/HCV-co-infected patients showed an increased frequency of HLA-C2/C2 in spontaneous clearance of HCV compared to chronic infected individuals but no association of KIR2DL3-HLAC1/C1 with spontaneous clearance of HCV [156]. The gene for the activating KIR receptor *KIR2DS3* was significantly higher in patients who resolved HCV infections in the presence of HLA-C2 [157]. *KIR/HLA-C* combinations also influence the development of HCV related hepatocellular carcinoma (HCC). A study of 787 chronic HCV individuals, with and without HCC, revealed an association of *KIR2DL2/HLA-C1* and *KIR2DS2/HLA-C1* and HCC in patients younger than 65 [158]. Hu et al. identified the combination of *KIR2DL2/HLA-C1* as a risk factor for chronic HCV infections and associated it with non-responders to PegIFN/RBV therapy [159]. In contrast, another study showed that *KIR2DL2*, *KIR2DS2*, *KIR2DL2/L3* were more frequent in subjects with HCV clearance, whereas *KIR2DL3/L3,* as well as *KIR2DL3/L3-HLA-C1* or *C1/C1* are associated with chronic HCV infection [160]. Previous research demonstrated that SNPs in the *HLA-C* gene can influence the outcome of HIV infections but little is known about the impact of SNPs in *KIR* genes on HCV progression. The analysis of four KIR/HLA-C SNPs in a high-risk Chinese population identified two SNPs (KIR2DS4/S1/L1 rs3544047-A and HLA-C rs1130838-A) that are associated with increased susceptibility to HCV infection [161]. A study about the connection of *KIR/HLA* genes and HCV in Romanian patients revealed that the expression of *KIR2DL3, KIR2DL5, KIR2DS4, KIR3DL3* genes and specific *HLA* alleles like HLA-A*23:01, -B*44:02 and -C*04:02 may increase the susceptibility of the patients to develop chronic HCV infection [162]. These inconsistent results may be due to the lack of allele-specific KIR genotyping in most studies. While the presence/absence of *KIR* genes in gene association studies may provide a first glimpse of the putative role in diseases control, low resolution of *KIR* genes may mask potential effects as high, as well as low binding KIR allotypes are lumped together.

As described for HIV, the presentation of viral peptides by HLA can modulate the binding to KIRs and affect NK cell function. Presentation of a core protein of HCV and binding to HLA-C*03:04 led to inhibition of KIR2DL3^+^ NK cells through an increased binding of HLA-C*03:04 to KIR2DL3 (Figure 4) [163].

### 4.3. Human Cytomegaloviruses (CMV)

Acute infection with CMV induces the expansion of NKG2C^+^ NK cell subsets, which can remain stable over several years [164]. Reactivation of CMV in patients after hematopoietic stem cell transplantation leads to an expansion of NKG2C^+^ KIR^+^ NK cells, which are potent producers of IFNγ during acute infection and also after clearance [165]. Moreover, multi-parametric flow cytometric analysis of NK cell subsets revealed that the immunological checkpoint molecule programmed death 1 (PD-1) is highly expressed on mature NK cells (CD56^dim^NKG2A^-^KIR^+^CD57^+^) cells of CMV-seropositive donors [166].

In comparison to CMV-seronegative individuals, healthy CMV-seropositive individuals exhibit a stable imprint in their KIR repertoire because of the expansion of NK cells expressing inhibitory KIRs specific for self-HLA-C. Individuals, homozygous for *HLA-C1* had an increased frequency for KIR2DL3, whereas *HLA-C2* homozygous donors had high frequencies of KIR2DL1 expressing NK cells. Moreover, the KIR phenotyping revealed an implication of activating KIRs (KIR2DS2, KIR2DS4 and KIR3DS1) in NK cell expansion [167,168]. In contrast, NKG2C^+^ NK cells lack the expression of the inhibitory NK cell receptors NKG2A and KIR3DL1 [169]. Co-culture experiments of NK cells with CMV-infected fibroblasts showed an increased expression of KIR2DL1, KIR2DL3 and KIR3DS1 in CMV-seropositive donors. In line with previous results, NKG2C^+^ NK cells had an increased expression of KIR2DL1 but not KIR3DL1 [168]. The adaptation of NKG2C^+^ NK cells is highly associated with the cenA-located C2-specific KIR2DL1, independent from the *KIR2DL1* allele. A correlation of NKG2C^+^ NK cells co-expressing one of the HLA-C specific KIR2DL1/L2/L3 with the CMV-specific IgG Ab concentration showed that the adaptation is only restricted to NK cells expressing KIR2DL1 [170]. A study with patients with hematological malignancies that were transplanted with NKG2C-/- umbilical cord blood showed an expansion of CD56^dim^NKG2A^-^NKG2C^-^KIR^+^ NK cells, mainly expressing KIR2DS1 and KIR3DS1 after CMV reactivation [171]. In connection with placental CMV infection, KIR2DS1^+^ decidual NK cells acquired a higher cytotoxic function when exposed to CMV-infected decidual stromal cells [172]. Co-culture experiments of NK cell subsets with CMV-infected human fetal foreskin fibroblasts activated KIR2DS1-expressing NK cells. Blocking with the pan-HLA class I antibody W6/32 had an influence on the KIR2DS1/HLA-C2 interaction but not on the interaction with KIR2DL1, indicating a different recognition of HLA-C by KIR2DL1 and KIR2DS1 [173].

Despite the elicited host immune response, generating a permanent phenotypical imprint in T and NK cell subsets, CMV stays persistent in the host for a lifetime. Like many other viruses, CMV developed numerous strategies to evade the host’s immune response. In order to avoid NK cell-mediated killing of infected cells, CMV promotes the expression of ligands that bind to inhibitory NK cell receptors and inhibits the expression of ligands that enable the activation of NK cells. Mechanisms for NK cell inhibition include the expression of a viral MHC class I-like protein UL18, which binds the LIR-1 inhibitory NK cell receptor, or the expression of the TRAIL death receptor [174,175]. Some CMV proteins and RNAs are directly involved in HLA class I antigen-presentation by down-modulating HLA-A and -B and to some extent also HLA-C on CMV-infected cells [176]. CMV gene products US3 and US6 downregulate HLA-C and HLA-G by two different mechanisms in human trophoblast [177], but they are resistant to degradation associated with US2 and US11 [178,179]. Other studies demonstrated that US2 is involved in HLA-A and -C downmodulation but not HLA-B, whereas US11 is able to downmodulate all three HLA class I molecules [180,181] (Figure 4).

The immune evasion strategy of CMV by affecting the antigen presentation of HLA class I molecules also influences the efficiency of HLA class I-restricted T cell response in an allotype-specific manner. HLA-C*07:02-restricted T cells are able to kill CMV-infected cells by recognizing the viral antigen IE-1 in a much more efficient way than HLA-A and -B-restricted T cells. At the same time, CMV-infected cells were resistant to NK cells carrying KIR2DL3 [54]. In line with that, CMV-specific T cells restricted by HLA-C*07:02 expand markedly with age, representing the dominant CD8^+^ T cell repertoire in people over the age of 70 years [182].

**Table 2:** HLA-C-mediated impact in HIV-1, HCV and CMV infections.

Mechanism/Observation	Virus	Reference
***KIR/HLA-C* disease association**		
High HLA-C expression is associated with HIV-1 control	HIV-1	[115,121,122,123]
KIR2DL3/HLA-C1 combination is associated with severe HIV-1 clinical outcome	HIV-1	[131,132,133]
KIR2DL3/HLA-C1 combination is associated with HIV-1 protection	HIV-1	[134,135]
Homozygous KIR2DL3/HLA-C1 combination is associated with spontaneous HCV resolution and better treatment response	HCV	[20,149,152,153]
Higher frequency of KIR2DL2/HLA-C1 in chronic HCV infection	HCV	[154]
Higher frequency of KIR2DL3 and low frequency of KIR2DL2 in persistent HCV infection	HCV	[3]
Increased frequency of C2/C2 in spontaneous HCV clearance	HCV/HIV-1	[156]
Higher frequency of KIR2DS3/HLA-C2 in HCV resolution	HCV	[157]
Combination of KIR2DL2/HLA-C1 is a risk factor for chronic HCV infecton and associated with no treatment response	HCV	[159]
Homozygous KIR2DL3/HLA-C1 is associated with chronic HCV infection, higher frequency of KIR2DL2, KIR2DS2 and KIR2DL2/L3 in HCV clearance	HCV	[142]
**Alterations of NK cell repertoire**		
Reactivation of CMV in patients with hemaotopoietic stem cell transplantation leads to expansion of NKG2C^+^ KIR^+^ NK cells	CMV	[165]
Healthy CMV-infected individuals have a stable imprint in the KIR repertoire with a bias for inhibitory KIRs specific for self HLA-C	CMV	[167]
Increased expression of KIR2DL1 in NKG2C^+^ NK cells in CMV infection	CMV	[168,170]
Mature CD56^dim^NKG2A^-^KIR^+^CD57^+^ NK cells of seropositive CMV donors highly express PD-1	CMV	[166]
**Antiviral activity of NK cells**		
KIR2DL1-3^+^ NK cells sense changes in HLA-C expression by increased antiviral activity	HIV-1	[141]
**Virus-mediated modulation of HLA-C**		
HIV-1 Vpu mediates HLA-C downmodulation	HIV-1	[140,143]
HIV-2 Vif mediates HLA-C downmodulation	HIV-1	[143]
Downmodulation of HLA-C by various CMV proteins	CMV	[176,177,180,181]
**Selection of viral peptides**		
KIR2DL2-associated HIV-1 sequence polymorphisms modulate NK cell function	HIV-1	[83]
HLA-C*0102-restricted HIV-1 p24 Gag epitopes modulates KIR2DL2 binding	HIV-1	[145]
Sequence polymorphismus in HIV-1 p24 Gag modulates binding of KIR2DL2/3 to HLA-C*0304	HIV-1	[19,146]
Reduced expression of HIV-1-derived peptides and HLA-C*1403 and HLA-C*1202	HIV-1	[130]
HIV-1-derived peptides reduce binding of HLA-C*0304 to KIR2DL3	HIV-1	[147]
Presentation of HCV core protein by HLA-C*0304 results in inhibition of KIR2DL3+ NK cells	HCV	[163]

### 4.4. Other Viruses

In addition to HIV-1, HCV and CMV infection, there are only a few reports about the role of HLA-C in other viral infections. Reasons for that could be that HLA class I expression and modulation are usually assessed by a pan-HLA class I antibody, which does not allow the differentiation of specific HLA-A, -B or -C expression that HLA-C was disregarded because of its low surface expression or thought not being modulated in viral infections. The following chapter briefly summarizes the information about KIRs and HLA-C in the context of other viral infections. 

One of the first reports of HLA-C downregulation of HLA-C expression in the context of viral infections was described by Elboim et al. The group showed that, upon herpes simplex virus type 2 infection (HSV-2), HLA-C is downregulated from the cell surface of dendritic cells by the viral protein ICP47, which induces killing of the infected cell by NK cells [183].

An in vitro influenza A infection model showed that KIR2DL3^+^ NK cells from homozygous *HLA-C1* donors responded more rapidly with IFNγ secretion and displayed greater degranulation than KIR2DL1^+^ NK cells from *HLA-C2* homozygous subjects [184]. Genetic association studies between KIR and influenza infection progression with the 2009 pandemic influenza A (H1N1) virus showed a higher frequency of KIR2DL2 and/or KIR2DL3 in combination with their cognate HLA-C1 ligand and KIR2DL1 without the presence of HLA-C2 ligands in patients with severe influenza infection [185]. Infection of HLA class I transduced cell lines with influenza A and B viruses revealed that HLA class I downregulation occurs across a range of HLA-A, -B and -C allotypes [186]. In contrast, Hantavirus-infected cells upregulate HLA class I molecules, including HLA-C on the cell surface. Infection with Hantavirus leads to an expansion of NKG2C^+^ NK cells, which express educated inhibitory KIRs [187]. Acute infection with Chikungunya virus (CHIKV) leads to a clonal expansion of NKG2C^+^KIR2DL2/L3^+^NK cells in direct association with the viral load [188]. Another study, which investigated the impact of *KIR/HLA* class I genotypes on the susceptibility to CHIKV and Dengue virus (DENV) infection, showed an increased frequency of *HLA-C2* homozygous CHIKV-infected individuals compared to DENV-infected and control individuals in combination with *KIR2DL1* and an association with susceptibility to CHIKV infection [189]. In Lassa virus infection, the presentation of HLA-C-restricted viral epitopes led to a stronger binding to KIR2DL2^+^ NK cells and inhibited NK cells [21]. Association studies of *KIR/HLA* in Ebola virus infected patients identified various KIRs that are associated with clinical outcome of Ebola: One study showed that the activating KIR2DS1 and KIR2DS3 are associated with a fatal outcome in Ebola infection [190]. Contrary to these findings, Wawina-Bokalanga identified *KIR2DL2* as a protective gene, whereas *KIR2DL5* and KIR2DS4*003 were more frequent in persons who died from Ebola infection [191]. A third study found an increased expression of KIR2DL1 on NK cells in Ebola-infected patients [192]. Although Maucourant et al. did not find differences in the expression profile of inhibitory KIRs on NK cells or NK cell education in COVID-19 patient compared to controls [193], there are a few reports about specific KIR/HLA-C combinations in COVID-19. HLA-C*05:01-restricted peptides promote binding and activation of KIR2DS4^+^ NK cells [194]. The gene combination of *KIR2DS2/HLA-C1* was more common in asymptomatic-paucisymptomatic patients compared to patients with severe symptoms [195]. 

## 5. Conclusions

HLA-C takes a special position in the regulation of NK cells. Its peculiar features affecting its expression and antigen presentation separate it from other classical molecules. Its unique role is further highlighted by the rapid co-evolution of HLA-C recognizing KIRs since its first appearance. HLA-C exerts an extraordinary role in pregnancy, as it is the only classical HLA class I gene expressed at the maternal-fetal interface. However, accumulating evidence shows that interactions between KIRs and HLA-C also impact the course of various pathological conditions, including infectious diseases. The diversity of KIRs and HLA-C leads to an extraordinary complexity of these interactions posing a challenge for researchers to grasp the impact of KIR/HLA-C interactions on NK cell function and on the course of human diseases. Integration of in vitro binding data, functional NK cell data, as well as high resolution gene association studies may provide the foundation for the generation of prediction models for the outcome of various diseases and the contribution of NK cells in those.

## Figures and Tables

**Table 1:** Distribution of HLA-C1 and -C2 allotypes.

HLA-C1 (80N)	HLA-C2 (80K)
01:02, 01:03, 01:04, 01:05	02:02, 02:03, 02:04, 02:05
03:02, 03:03, 03:04, 03:05, 03:06,03:08, 03:09 03:10, 03:11, 03:12, 03:13, 03:14	03:07
	04:01, 04:03, 04:04, 04:05, 04:06, 04:07, 04:08
	05:01, 05:02, 05:03, 05:04
	06:02, 06:03, 06:04, 06:05, 06:06, 06:07
07:01, 07:02, 07:03, 07:04, 07:05, 07:06, 07:08, 07:10, 07:11, 07:12, 07:13, 07:14, 07:15	07:07, 07:09
08:01, 08:02, 08:03, 08:04, 08:05, 08:06, 08:07, 08:08, 08:09	
12:02, 12:03, 12:06, 12:08	12:04, 12:05, 12:07
13:01	
14:02, 14:03, 14:05	14:04
15:07	15:02, 15:03, 15:04, 15:05, 15:06, 15:08, 15:09, 15:10, 15:11
16:01, 16:04	16:02
	17:01, 17:02, 17:03
	18:01, 18:02

Based on a dimorphism at position 80 of the α1 domain, HLA-C molecules can be subdivided into two groups: HLA-C group 1 and group 2 [80,87].
